# Evolution of dislocation microstructure in irradiated Zr alloys determined by X-ray peak profile analysis

**DOI:** 10.1107/S1600576720015885

**Published:** 2021-02-01

**Authors:** István Groma, Ildikó Szenthe, Éva Ódor, Bertalan Jóni, Gyula Zilahi, Zoltán Dankházi, Gábor Ribárik, Zoltán Hózer

**Affiliations:** a Department of Materials Physics, ELTE, Pázmány Péter sétány 1/A, Budapest, 1117, Hungary; b MTA EK, Konkoly Thege Miklós út 29-33, Budapest, 1121, Hungary

**Keywords:** nuclear materials, X-ray line profile analysis, dislocation density, neutron irradiation, vacancies, interstitial atoms

## Abstract

During neutron irradiation of metals, owing to the enhanced number of vacancies and interstitial atoms, the climb motion of dislocations becomes significant at room temperature, leading to a recrystallization of the material. Moreover, the vacancies and interstitial atoms tend to form prismatic dislocation loops that play a crucial role in the plastic properties of the materials. X-ray peak profile analysis is an efficient nondestructive method to determine the properties of dislocation microstructure. In the first half of this article, the foundation of the asymptotic peak broadening theory and the related restricted-moments peak-evaluation method is summarized. After this, the microstructural parameters obtained by X-ray peak profile analysis are reported for irradiated E110 and E110G Zr alloys used as cladding material in the nuclear industry.

## Abstract 2

The dislocation microstructure developing during neutron irradiation is determined by X-ray line profile analysis.

## 1. Introduction   

One of the constant objectives of various technical and economic improvements related to nuclear reactors is the sustainable and increasingly safe operation of existing reactors. Increasing safety requirements are a constant challenge in optimizing the properties of reactor materials.

During the operation of nuclear reactors, radioactive fission products are produced in the fuel elements. These fission products can produce radiation dangerous to living organisms. Therefore, the most important task of the fuel-element cladding is to act as a primary barrier to the release of radioactive isotopes from the fuel rods into the primary coolant and potentially to the environment. Even under extreme operating conditions, it is necessary to preserve the integrity of the cladding tubes.

Further developments are needed to improve the mechanical properties of the fuel cladding to increase its radiation and corrosion resistance during normal and extreme operating conditions for enhancement of the electricity production and to provide significant economic benefits.

This requires one to perform and evaluate a large number of fuel-behavior simulations covering all reactor states under any given circumstances in order to understand the expected behavior of new fuel elements during operation. These simulations must be carried out with validated computer programs supported by experimental results.

In nuclear applications, to meet the requirement of the material for cladding tubes, two types of zirconium binary alloys have been used until recently, in which tin and niobium are the main alloying elements. Nowadays, ternary system alloys formed from a combination of these elements are being developed for some reactor designs. Zirconium and its alloys satisfy the requirements for nuclear-fuel cladding: low thermal neutron absorption cross section, high resistance to irradiation, good corrosion resistance in high-temperature and high-pressure steam or water, and adequate mechanical properties.

Detailed transmission electron microscopy (TEM) investigations have revealed that the self-interstitial atoms (SIAs) and vacancies formed during neutron irradiation tend to form prismatic dislocation loops that play a crucial role in the mechanical properties of the cladding material [for a comprehensive review see Griffiths (1988 ▸)]. Beside TEM, X-ray peak profile analysis is an efficient method to determine the microstructure developing during the service of the cladding material. In a recent study, Seymour *et al.* (2017 ▸) applied the convolutional multiple whole profile (CMWP) fitting method to evaluate the intensity distribution measured on neutron-irradiated Zr alloys.

In the present article, X-ray peak profile measurements are reported on neutron-irradiated Zr samples. In the first part of this article, the theoretical background of the asymptotic single peak profile analysis (APA) is briefly summarized. The main difference between the CMWP and APA methods is that APA does not require any assumption about the microstructure before the evaluation of the peaks. In the second half of this article, the results of the peak profile analysis obtained on heat-treated and neutron-irradiated Zr alloys applied for cladding tubes are reported.

## 2. X-ray peak profile analysis   

In X-ray peak profile measurement, the shape of a dedicated Bragg peak is determined with a high angular precision. In order to reduce the peak broadening related to the different instrumental effects (called instrumental peak broadening), like the finite width in wavelength of the X-ray radiation produced by the X-ray generator, a special double-crystal diffractometer should be applied with an appropriate monochromator (see below for technical details).

### 2.1. Strain broadening   

In general, the intensity distribution *I*(**s**) is a 3D function, where **s** is the scattering vector measured from the diffraction vector **g** corresponding to the Bragg reflection selected. In a common setup called ‘strain broadening’, by the appropriate swinging of the sample, the 3D intensity distribution is integrated for the plane perpendicular to **g**. With this one measures the 1D *I*(*q*) intensity distribution with 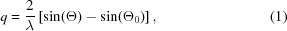
where λ is the wavelength of the X-rays, Θ is the scattering angle and Θ_0_ is the Bragg angle of the reflection used. The peak broadening is typically of the order of 0.1°.

### 2.2. Different sources of peak broadening   

One of the main sources of peak broadening is the finite crystalline size. Note that this is not necessarily the actual grain size. If owing to deformation the grains subdivide into subgrains with larger than ∼0.1° misorientation angle in between the subgrains, the broadening is determined by the subgrain size. For distinguishing between the real and the subgrain sizes, those that can actually be determined by peak profile analysis, the term ‘coherent domain size’ is commonly used.

The peak broadening caused by the finite coherent domain size is determined by the reciprocal of the characteristic domain size (Wilson, 1962 ▸). The broadening can be well described by a Lorentzian function with half-width inversely proportional to the domain size *d* (Wilson, 1962 ▸; Borbély & Groma, 2001 ▸). As explained below for the evaluation method applied here, the asymptotic decay of the peaks plays a crucial role. For a Lorentzian function, the intensity distribution decays as 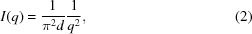
where *d* is the average coherent domain size. The above expression is valid only if the peak is normalized to 1, *i.e.*

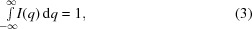
and *q* is measured from the center of gravity of the peak, 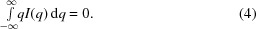
Local lattice-parameter variation generated by internal stress also leads to peak broadening. The first theory of peak broadening caused by internal strain was developed by Warren & Averbach (1950 ▸, 1952 ▸). However, since they did not consider singular stress sources, like dislocations, the evaluation method they proposed is not suitable to analyze peaks measured on samples containing dislocations. The first theory of peak broadening caused by dislocation was developed by Krivoglaz (1969 ▸). However, since he considered only dislocations randomly placed in the crystal, owing to the 1/*r* type of the stress field of dislocations (where *r* is the distance from the dislocation line), the broadening depends on a system size that has never been observed experimentally. The problem has been formally solved by Wilkens (1969 ▸, 1970 ▸) by introducing the concept of ‘restricted random’ dislocation distribution meaning that the dislocations are randomly placed only in areas with a given size. The problem with the concept is that there is no experimental or numerical evidence that the dislocation–dislocation correlation follows this rather specific feature.

In the 1980s, Groma and co-workers proposed a general theory of peak broadening caused by dislocations (Groma, 1998 ▸; Borbély & Groma, 2001 ▸; Groma & Borbély, 2004 ▸). They showed that the intensity distribution asymptotically decays as 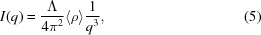
where 〈ρ〉 is the dislocation density, and Λ is a constant depending on the diffraction and the Burgers and line direction vectors of the dislocation (see below for further details). The quantity Λ is commonly written in the form 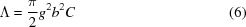
(Groma, 1998 ▸; Borbély & Groma, 2001 ▸; Groma & Borbély, 2004 ▸), where *C* is called the ‘contrast factor’, *g* is the diffraction vector magnitude and *b* is the Burgers vector magnitude. A remarkable feature of the above results is that the tail is not affected by the dislocation–dislocation correlation. The actual correlation properties play a role only in the shape of the center part of the peaks. For small enough Fourier parameter *L*, the Fourier transform of the intensity distribution is (Groma, 1998 ▸; Groma & Borbély, 2004 ▸) 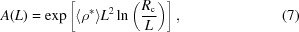
where 〈ρ*〉 = Λ〈ρ〉 is commonly called the ‘formal’ dislocation density and *R*_c_ is a parameter with length dimension determined by the dislocation–dislocation correlation.

### 2.3. Restricted moments   

In order to determine the coherent domain size and the dislocation density from the measured intensity distribution, one should fit the sum of the functions given by equations (2) and (5), 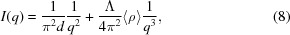
to the asymptotic part of the intensity distribution. However, since in the tail of the measured *I*(*q*) the relative noise of the data points is often relatively large and one does not really know in advance from which *q* value the asymptotic expression is valid, the fitting cannot be easily performed.

To improve the accuracy of the parameter determination it is useful to consider the ‘integrated’ quantities called ‘restricted moments’, defined as (Groma, 1998 ▸; Borbély & Groma, 2001 ▸; Groma & Borbély, 2004 ▸) 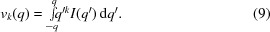
One can easily see from equation (8) that for large enough *q* values the second-order restricted moment reads as 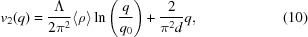
where *q*_0_ is a parameter determined by the dislocation–dislocation correlation (Groma, 1998 ▸; Borbély & Groma, 2001 ▸; Groma & Borbély, 2004 ▸).

It is often useful to consider the quantity 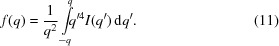
In the asymptotic regime it reads as 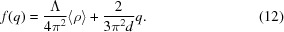
For large enough *q* values, *f*(*q*) is linear in *q* with a slope inversely proportional to the coherent domain size and with an intercept proportional to the dislocation density. According to our experience, if the domain size is larger than ∼1 µm, in the *q* regime accessible by a common experimental setup, the function *f*(*q*) tends to a constant, which means the coherent domain size cannot be determined. It does not contribute to the peak broadening in the *q* regime that one can reach. Note also that the measured profiles often contain a background that one has to subtract before the evaluation of the peak. The actual level of background, however, is often not easy to determine. If one analyzes both *v*_2_(*q*) and *f*(*q*) the two should provide the same parameters if the background level is chosen appropriately. So, analyzing the two restricted moments provides the possibility of an ‘internal’ checking of the background selection.

## 3. Experimental investigations   

### 3.1. Samples investigated   

Zirconium–niobium alloys are used as the material of the cladding tubes in the VVER-440 model V213 type pressurized water reactors. Measurements were performed on two types of materials used for cladding tubes, named as E110 and E110G. The Russian manufacturer of the E110 cladding material is switching from an earlier electrolytic process to metal ‘sponge’ technology. In the currently used E110 cladding alloy, the zirconium is produced 60% through an iodide process and 40% through an electrolytic process. In the new E110G cladding, 70% of the zirconium metal comes from this ‘sponge’, with the residual 30% made by the iodide process. The main chemical composition of E110G alloy remains the same, 99% zirconium and 1% niobium, but permissible levels of certain trace element concentrations are upgraded (Nikulin *et al.*, 2011 ▸).

The X-ray diffraction measurements were carried out on samples taken from Zr alloy tubes, E110 and E110G. Measurements were performed on the as-received and neutron-irradiated samples. The irradiation was performed in the BAGIRA (Budapest advanced gas-cooled irradiation rig with Al structure) irradiation rig in the Budapest Research Reactor. This allowed us to perform irradiation with different fluences at a given temperature. The radiation damage of the samples has been tested in the connecting hot laboratory.

To simulate the different burnup states, the samples were irradiated with neutrons at two different fast-neutron-flux locations of the reactor core for 1675.5 h. During the entire irradiation period, one sample series received a fast neutron fluence of 1.99 × 10^24^ nm^−2^ (low fluence) and the other recieved 3.2 × 10^24^ nm^−2^ (high fluence) (*E* > 1.5 MeV). The ‘low fluence’ corresponds to ∼0.4 dpa while the ‘high fluence’ is ∼0.7 dpa. During the irradiation, the average temperature of the sample series receiving the ‘low fluence’ was in the range of 533 ± 10 K, while the other sample series was in the range of 573 ± 10 K. In this study we aim to investigate the early stage of radiation damage, so the neutron fluences applied are smaller than in most of the investigations presented earlier (Jostsons *et al.*, 1977 ▸; Griffiths, 1988 ▸; Seymour *et al.*, 2017 ▸).

Since during the irradiation the samples were held at a temperature of ∼553 ± 20 K for a long time, it was important to see if any recovery process could take place even without neutron irradiation just because of the heat treatment at a relatively elevated temperature. So, non-irradiated E110G samples were heat treated for 24 and 200 h at 533 ± 10 K in a vacuum chamber.

### 3.2. Experimental method   

The profile measurements were performed with a fine-focus Cu anode X-ray generator at 40 kV and 30 mA with wavelength λ = 0.15406 nm. In order to reduce the instrumental broadening, the symmetrical 220 reflection of a Ge monochromator was used. The *K*α_2_ component of the Cu radiation was eliminated using a 0.1 mm slit between the source and the Ge crystal. The profiles were registered by a linear position-sensitive DECTRIS MYTHEN2 R detector with 50 µm spatial resolution and 1280 channels. The sample–detector distance was 0.7 m, resulting in an angular resolution of the order of 0.004°.

Owing to the fabrication process, the tubes are strongly textured. The 〈*c*〉 axis is close to the radial direction with a small incline in the (*r* − θ) plane (Schemel *et al.*, 1989 ▸). So, peak profile measurements could be performed at the 0002 and 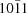 reflections of Zr. (The intensities at the other peaks were not enough to evaluate the peaks.) To study the activated samples, the X-ray diffractometer assembly has been installed in the area of the Budapest Research Reactor that is licensed for radioactive material activities.

**Figure 1:**
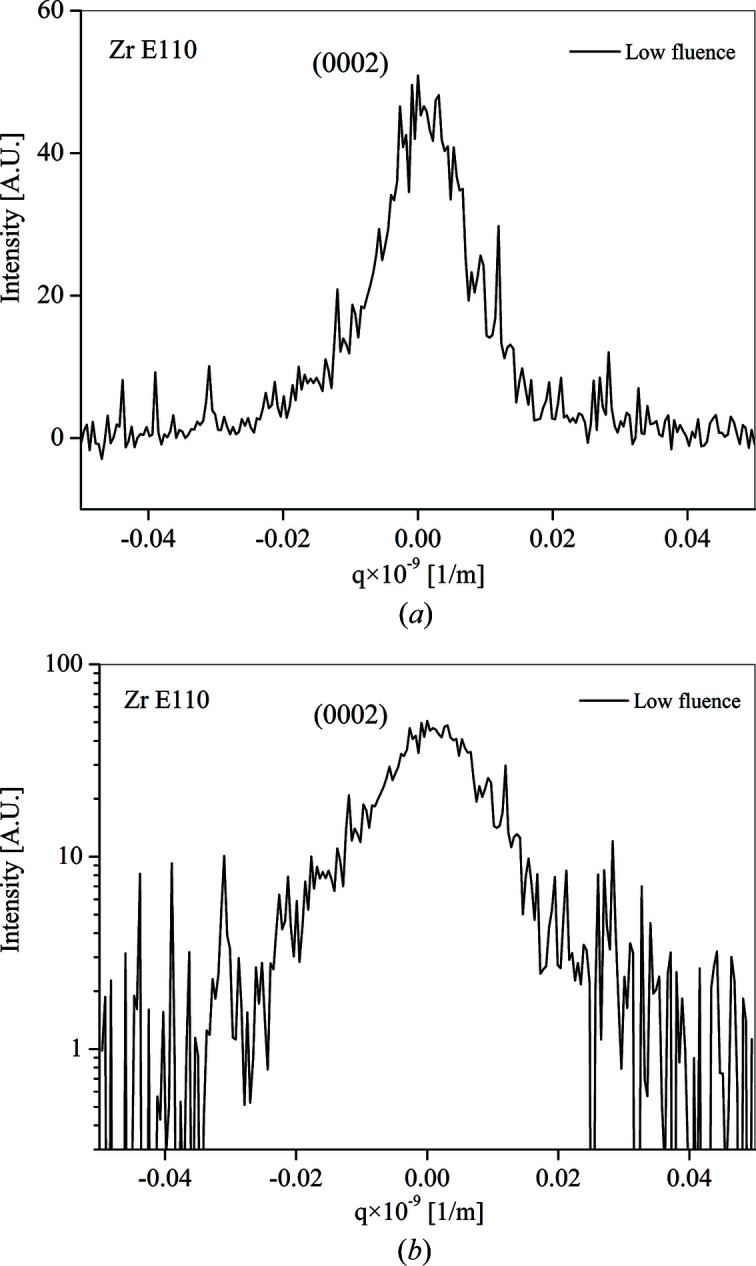
The intensity distribution at Bragg reflection 0002 with linear and logarithmic scales obtained for the sample E110 irradiated by the fluence 1.99 × 10^24^ nm^−2^.

The measured intensity distribution and the corresponding *v*_2_(*q*) and *v*_4_(*q*)/*q* ^2^ plots obtained for the sample E110 irradiated by the fluence of 1.99 × 10^24^ nm^−2^ (low fluence) are plotted in Figs. 1 ▸ and 2 ▸, respectively.

**Figure 2:**
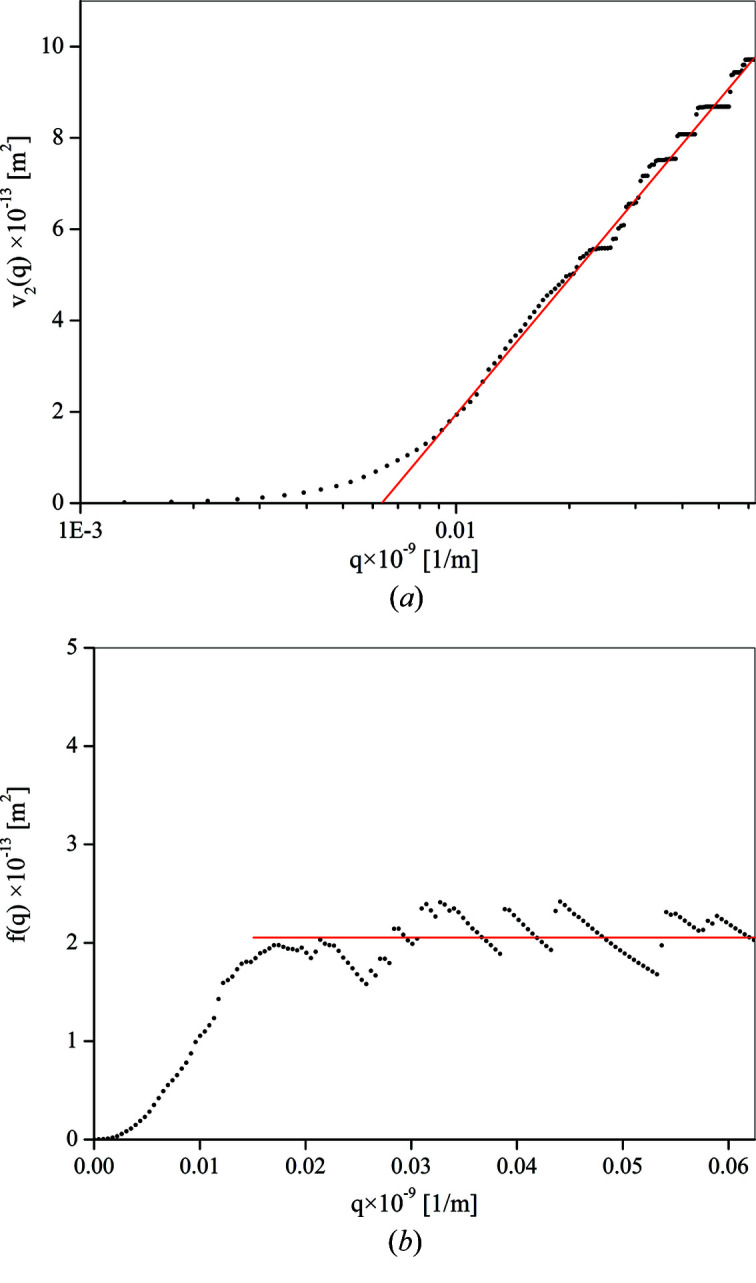
The *v*_2_(*q*) (top box) and *v*_4_(*q*)/*q* ^2^ (bottom box) restricted moments at Bragg reflection 0002 obtained for the sample E110 irradiated by the fluence 1.99 × 10^24^ nm^−2^.

**Figure 3:**
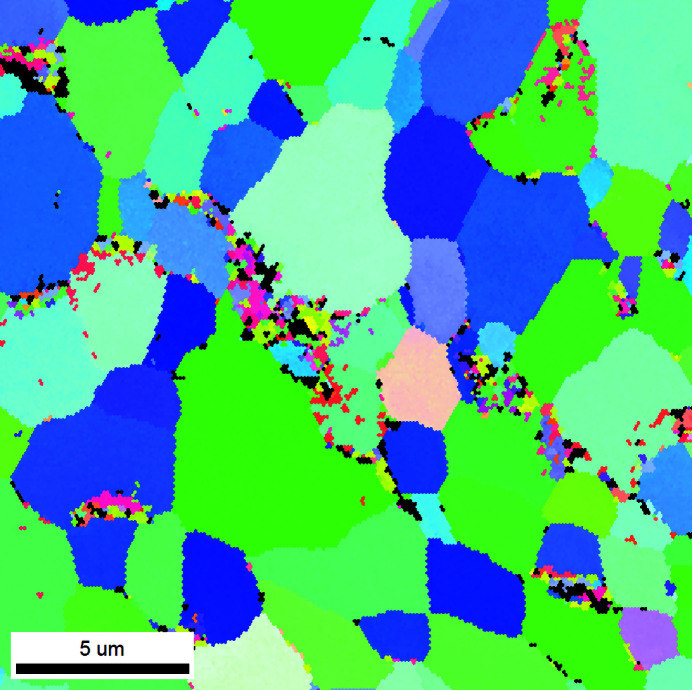
The EBSD inverse pole figure map obtained for the non-irradiated E110 sample.

As seen in Fig. 2 ▸, the asymptotic part of *v*_2_(*q*) is linear in ln(*q*) and *v*_4_(*q*)/*q* ^2^ tends to a constant. According to equations (10) and (12), these indicate that the coherent domain size is definitely larger than 1 µm (in the asymptotic regime the slope of the *v*_4_(*q*)/*q* ^2^ versus *q* plot is practically zero). So, for the samples investigated, the finite size broadening is negligible. This is supported by the electron backscatter diffraction (EBSD) inverse pole figure map, seen in Fig. 3 ▸, obtained on the same sample.

Although the signal-to-noise ratio in the intensity distributions measured is only of the order of 100 (for a deformed single crystal it can be as high as 10^5^), the formal dislocation density 〈ρ*〉 that one can obtain from the evaluation of the two restricted moments can be determined with a high accuracy. From the scatter of the data points in the *v*_2_(*q*) and *f*(*q*) plots, the estimated error is less than 5%.

## 4. Results   

### 4.1. Role of heat treatment   

Let us first analyze the non-irradiated samples subject to heat treatment at 533 ± 10 K.

**Figure 4:**
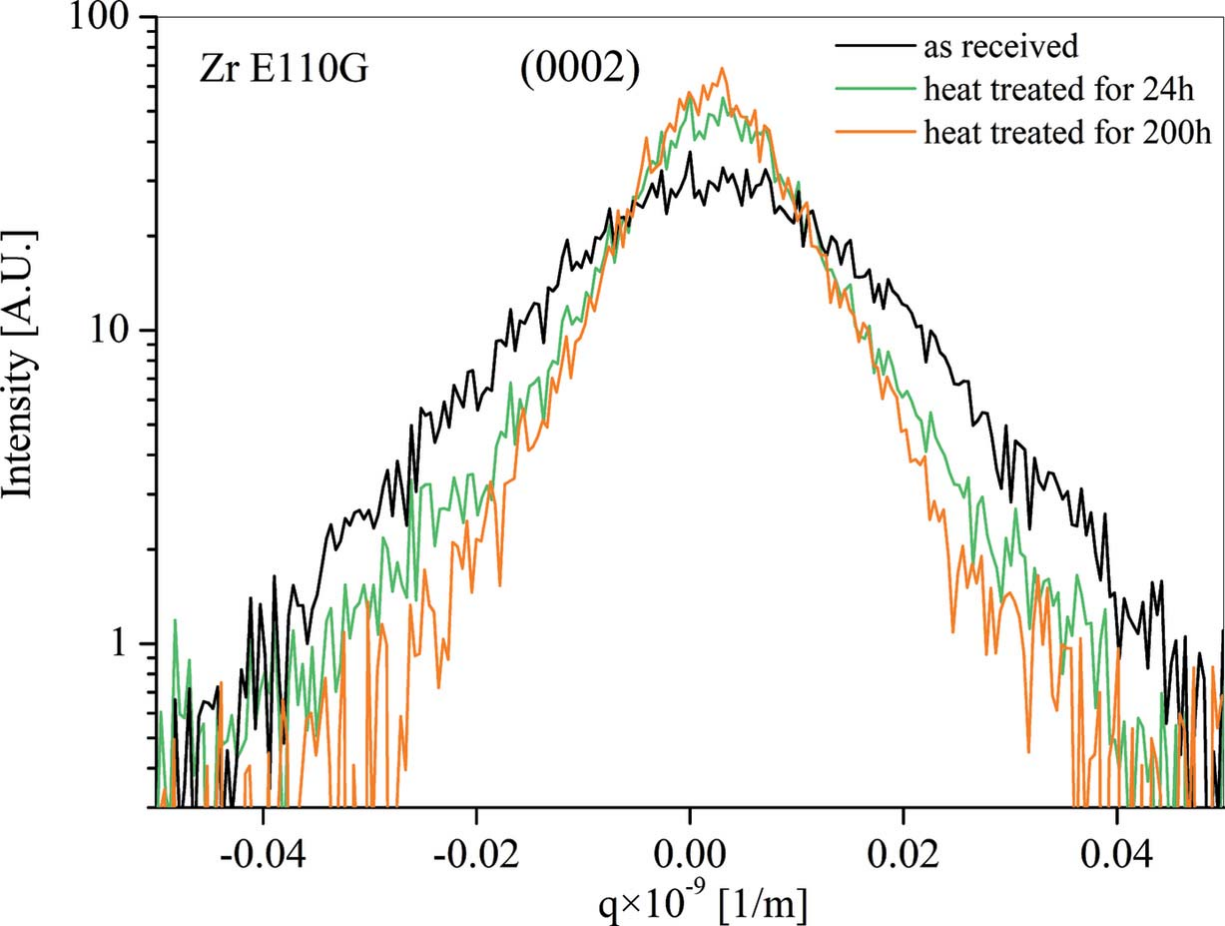
The intensity distributions of the 0002 reflection obtained for the as-received, heat treated for 24 h and heat treated for 200 h at 533 ± 10 K samples.

The peak profiles measured on the three samples (as received, heat treated for 24 h and heat treated for 200 h) are plotted in Fig. 4 ▸. The formal dislocation densities (〈ρ*〉) obtained from the restricted moments *v*_2_(*q*) and *v*_4_(*q*) are summarized in Table 1 ▸. For each sample, the two restricted moments give the same formal dislocation density within 10%. However, owing to the different contrast factors *C*, the values for the two reflections measured are different. Since during the manufacturing of the cladding tube the grains in the materials undergo a complex deformation it is assumed that the possible dislocation types in the different slip systems are activated with equal probability. This assumption was made owing to its simplicity, and its specifics do not change qualitatively the conclusions of the presented work. With this assumption one can use an average contrast factor 〈*C*〉. With the *ANIZC* program developed to calculate the contrast factor for any material (Borbély *et al.*, 2003 ▸), we obtained that for the two reflections measured 〈*C*_(0002)_〉 = 0.1758 and 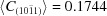. The dislocation densities obtained with these contrast factors are given in Table 2 ▸.

It is seen that the X-ray peaks become narrower with increasing heat-treatment time. This is reflected in the 〈ρ〉 values. The dislocation density goes down by about a factor of three after 200 h of heat treatment at 533 ± 10 K. In agreement with this, the Vickers hardness (Hv) of the material also reduced after the heat treatment. For the as-received sample, Hv = 1940 MPa, while after 200 h treatment, Hv = 1460 MPa (the values were measured at 5 N load).

The ratio of the formal dislocation densities obtained from the two reflections are somewhat different for the as-received and the heat-treated states (in Table 2 ▸ the average values calculated from the two reflections measured are given). This indicates that during the annihilation some dislocation dipoles can annihilate faster, meaning that the assumption of equal slip system population applied for the contrast-factor calculation is not exactly valid for the heat-treated states.

The results obtained indicate that the dislocations generated during the fabrication of the cladding tube recover within a relatively short time during the service of the tube. The rate of the annihilation of dislocations (requiring a non-conservative climb type of motion of the dislocations) can be considerably enhanced by the presence of the vacancies generated by the neutron irradiation. So, the annihilation of the initial dislocation density is even faster under service condition than in the case of the heat treatment performed. It can be assumed that the dislocations formed during the cladding-tube manufacturing are practically completely annihilated by the end of the neutron irradiation applied in our study.

### 4.2. Role of neutron irradiation   

**Figure 5:**
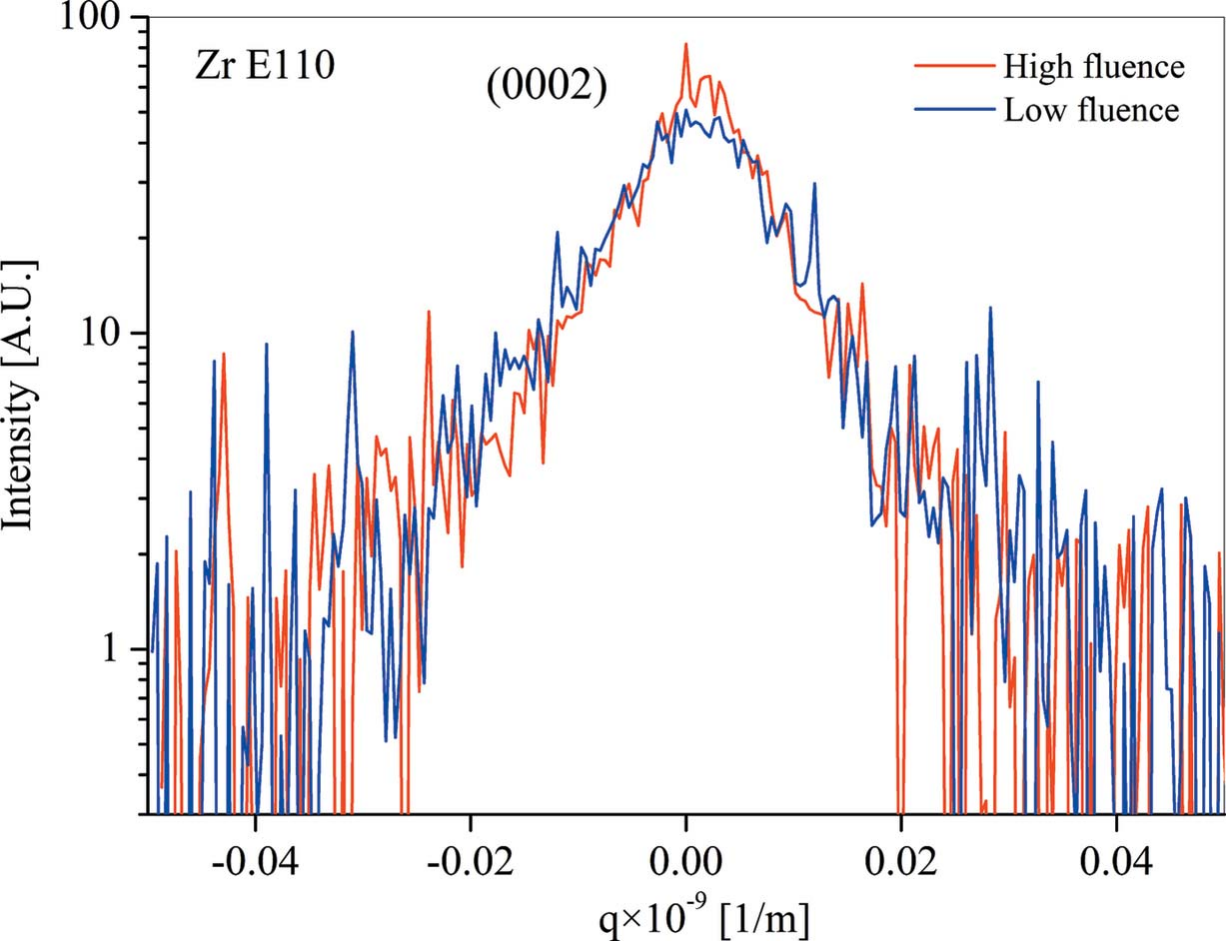
The intensity distributions obtained for the E110 samples irradiated with fluences 1.99 × 10^24^ nm^−2^ (low fluence) and 3.2 × 10^24^ nm^−2^ (high fluence) with the 0002 reflection.

The intensity distributions obtained for the E110 samples irradiated with fluences 1.99 × 10^24^ and 3.2 × 10^24^ nm^−2^ at the 0002 reflection are plotted in Fig. 5 ▸. (The intensity distributions for the 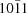 reflection and for the E110G samples show qualitatively the same features.) The 〈ρ*〉 values calculated from the restricted moments *v*_2_(*q*) and *v*_4_(*q*) are listed in Table 3 ▸ for the E110 materials and in Table 4 ▸ for the E110G materials.

Remarkably and somewhat surprisingly, for both materials the formal dislocation densities are smaller for the samples irradiated by larger fluences. A possible explanation is discussed below.

## 5. Discussion   

Owing to the neutron irradiation, vacancies and SIAs are formed. At somewhat higher fluence, the vacancies and SIAs tend to form disc-like clusters lying in one of the crystallographic planes of the Zr lattice (Griffiths, 1988 ▸). They can be considered as immobile prismatic dislocation loops. If the loop plane is in the basal plane of the Zr crystal, the loop is called 〈*c*〉 type. If the loop plane is in a non-basal plane perpendicular to the basal plane, the loop is called 〈*a*〉 type. The most common Burgers vector/plane-normal vector combinations are 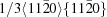, 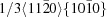 and 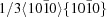 (Griffiths, 1988 ▸). If the plane normal has both 〈*a*〉 and 〈*c*〉 components the loop is called 〈*a* + *c*〉 type. The most important ones are 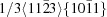 and 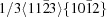.

The early stages in the formation of dislocation loops have recently been investigated computationally (Varvenne *et al.*, 2014 ▸; Dai *et al.*, 2017 ▸; Christensen *et al.*, 2015 ▸). It was found that both vacancies and SIAs diffuse anisotropically. As a result, 〈*a*〉-type loops can form more easily. This is in agreement with TEM results (Griffiths, 1988 ▸).

In order to determine the real dislocation density (not only the formal one given above), the contrast factors of the different possible prismatic loops have to be determined. In a detailed investigation, Balogh *et al.* (2016 ▸) numerically calculated the average contrast factors for different possible loops. The calculation was based on the *ANIZC* software package (Borbély *et al.*, 2003 ▸). In our analysis, it is assumed that each possible loop occurs with the same probability, leading to 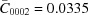 and 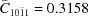 for 〈*a*〉-type loops, and 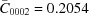 and 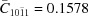 for 〈*c*〉-type loops. With these values, the average dislocation density and the ratio of the 〈*a*〉- and 〈*c*〉-type loops can be determined from the formal dislocation-density values given in Tables 3 ▸ and 4 ▸. The results are summarized in Table 5 ▸.

As confirmed earlier by TEM (Griffiths, 1988 ▸) and numerical simulations (Varvenne *et al.*, 2014 ▸; Dai *et al.*, 2017 ▸; Christensen *et al.*, 2015 ▸), in the fluence regime applied the loops are predominantly 〈*a*〉 type. For the E110 materials, the ratio of 〈*c*〉-type loops increases with increasing fluence. For the E110G material, it remains practically unchanged. Remarkably, however, for both materials the dislocation density is smaller for the samples irradiated with higher fluence. To understand this, we have to take into account that the dislocation density is the total circumference of the loops in a unit volume. So, if one takes a given total area of loops [*i.e.* a fixed number of SIAs (or vacancies) in the loops] the total circumference of the loops can be reduced if the diameter of the loops is increased. At the same time, the total energy of the system is obviously smaller if the total circumference of the loops is smaller. As a result, it is an energetically favorable process if the SIAs (or vacancies) from smaller loops move to larger loops. The process is similar to what is called Ostwald ripening in alloys. The ripening process is controlled by diffusion that in our case is enhanced by the pressure gradient generated by the dislocation loops and by the ‘individual’ vacancies generated by the irradiation. The ripening process may be enhanced by the somewhat higher temperature the ‘high fluence’ sample was subjected to during the irradiation. So, the results obtained indicate that during the irradiation the total number of SIAs (or vacancies) may increase, but owing to the ripening of the loops the dislocation density decreases. This has an important consequence in the mechanical properties of the cladding tubes. Since the prismatic loops are immobile objects they act as obstacles in the motion of mobile dislocations generated by the stress in the cladding tubes. Owing to the ripening process, the number of obstacles is reduced, resulting in a softening of the tubes. This is in agreement with the TEM results on the evolution of the loop diameter during post-irradiation heat treatment on neutron-irradiated zirconium alloys (Ribis *et al.*, 2010 ▸).

## 6. Conclusions   

X-ray peak profile measurements were performed on E110 and E110G Zr alloys irradiated by neutrons with two different fluence levels. The peaks were evaluated with the restricted-moment method. It was shown that the dislocation density corresponding either to dislocations generated during the manufacturing of the cladding tube or to prismatic dislocation loops formed during neutron irradiation can be determined with a high precision. The mobile dislocations annihilate within a few hundred hours at 533 K. In agreement with earlier TEM and computer-simulation results, the majority of the prismatic loops are 〈*a*〉 type. An important feature of the evolution of the prismatic loops formed during irradiation is that with a ripening-type process the smaller loops dissolve and the SIAs (or vacancies) move into the larger loops. 

## Figures and Tables

**Table 1:** The 〈ρ*〉 formal dislocation-density values obtained for the as-received, heat treated for 24 h and heat treated for 200 h at 260 ± 10°C samples, at reflections (0002) and 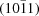 Values obtained from both the *v*_2_(*q*) and *v*_4_(*q*) restricted moments are given.

〈ρ*〉 (10^14^ m^−2^)	*v*_2_ (0002)	*v*_4_ (0002)	*v*_2_ 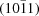	*v*_4_ 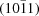
As received	29	26	30	29
Heat treated for 24 h	16	15	22	21
Heat treated for 200 h	9	8.6	12	12

**Table 2:** The 〈ρ〉 dislocation-density values obtained for the as-received, heat treated for 24 h and heat treated for 200 h at 533 ± 10 K samples

Sample E110G	〈ρ〉 (10^14^ m^−2^)
As received	27
Heat treated for 24 h	20
Heat treated for 200 h	9

**Table 3:** The 〈ρ*〉 formal dislocation-density values obtained for the E110 samples irradiated with fluences 1.99 × 10^24^ nm^−2^ (low fluence) and 3.2 × 10^24^ nm^−2^ (high fluence) Values obtained from the *v*_2_(*q*) and *v*_4_(*q*) restricted moments are given.

〈ρ*〉 (10^14^ m^−2^)	*v*_2_ (0002)	*v*_4_ (0002)	*v*_2_ 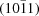	*v*_4_ 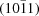
Low fluence	8.5	8.1	23	21
High fluence	6.7	6.5	15	12

**Table 4:** The 〈ρ*〉 formal dislocation-density values obtained for the E110G samples irradiated with fluences 1.99 × 10^24^ nm^−2^ (low fluence) and 3.2 × 10^24^ nm^−2^ (high fluence) Values obtained from the *v*_2_(*q*) and *v*_4_(*q*) restricted moments are given.

〈ρ*〉 (10^14^ m^−2^)	*v*_2_ (0002)	*v*_4_ (0002)	*v*_2_ 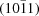	*v*_4_ 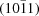
Low fluence	14	13	29	26
High fluence	6.8	6.5	15	11

**Table 5:** The 〈ρ〉 dislocation-density values and the fractions of the 〈*a*〉 and 〈*c*〉 type loops obtained for the irradiated E110 and E110G samples

Sample	〈ρ〉 (10^14^ m^−2^)	Fraction of 〈*a*〉 (%)	Fraction of 〈*c*〉 (%)
E110 low fluence	34	83	17
E110 high fluence	20	75	25
E110G low fluence	40	74	26
E110G high fluence	18	73	27
